# Biodegradable porous micro/nanoparticles with thermoresponsive gatekeepers for effective loading and precise delivery of active compounds at the body temperature

**DOI:** 10.1038/s41598-022-15069-x

**Published:** 2022-06-28

**Authors:** Kamonchanok Thananukul, Chariya Kaewsaneha, Pakorn Opaprakasit, Nadia Zine, Abdelhamid Elaissari

**Affiliations:** 1 School of Bio-Chemical Engineering and Technology, Sirindhorn International Institute of Technology (SIIT), Thammasat University, Pathum Thani, 12121 Thailand; 2 Univ Lyon, University Claude Bernard Lyon-1, CNRS, ISA-UMR 5280, 69622 Villeurbanne, France

**Keywords:** Drug delivery, Materials science, Polymer chemistry

## Abstract

Stimuli-responsive controlled delivery systems are of interest for preventing premature leakages and ensuring precise releases of active compounds at target sites. In this study, porous biodegradable micro/nanoparticles embedded with thermoresponsive gatekeepers are designed and developed based on Eudragit RS100 (PNIPAM@RS100) and poly(N-isopropylacrylamide) *via* a double emulsion solvent evaporation technique. The effect of initiator types on the polymerization of NIPAM monomer/methylene-bis-acrylamide (MBA) crosslinker was investigated at 60 °C for thermal initiators and ambient temperature for redox initiators. The crosslinked PNIPAM plays a key role as thermal-triggered gatekeepers with high loading efficiency and precise release of a model active compound, Nile Blue A (NB). Below the volume phase transition temperature (T_VPT_), the gatekeepers possess a swollen conformation to block the pores and store NB within the cavities. Above its T_VPT_, the chains rearrange, allowing gate opening and a rapid and constant release rate of the compound until completion. A precise “on–off” switchable release efficiency of PNIPAM@RS100 was demonstrated by changing the temperatures to 4 and 40 °C. The materials are a promising candidate for controlled drug delivery systems with a precise and easy triggering mechanism at the body temperature for effective treatments.

## Introduction

Smart delivery systems have attracted growing attention for selective delivery of active compounds, especially therapeutic agents, to targeted disease tissues. Smart drug delivery carriers are designed to deliver drugs without premature leakage and rapidly release loaded drugs after being uptaken by internal and external stimuli^1^. Stimuli-responsive drug delivery systems have crucial functions in responding to biological environment stimuli, such as pH^2^, temperature^3^, electric or magnetic field^4^, light^5^, enzyme^6^, and biological stimuli^7^. These systems can be applied externally and remotely to achieve controlled release from the drug carriers.

Various micro- and nano-particles have been employed in biomedical/pharmaceutical fields for effective treatments by improving the bioavailability and longevity of specific therapeutic in the blood system and increasing its accumulation at the biological target (thus reducing side effects)^8^. Microparticles are typically useful as reservoir systems for the controlled release of drugs, including polypeptide/protein drugs. At the same time, nanoparticles are suitable for intravenous injection administration, aiming at a cellular/subcellular target^9,10^. Chen et al*.* reported that large-sized microparticles of gefitinib-loaded poly(lactic-*co*-glycolic acid) (PLGA) showed a prolonged release mechanism^11^. Kim et al*.* fabricated highly porous PLGA particles (11.5 ± 0.4 µm) loaded with tumor-drug for lung cancer treatment *via* an inhalation route^12^. The PLGA-based microparticles gradually released the drugs over 7 days. Pulmonary administration of the microparticles resulted in their deposition in mouse lungs and remained for up to a week. Reczyńska et al*.* reported that microparticles with diameters between 2 and 3.5 µm with a narrow size distribution could increase the effectiveness of their deposition in deeper parts of the respiratory tract^13^. Nanoparticles provide additional advantages in precisely delivering active compounds to a specific target. This is particularly of interest as chemotherapy drug carriers. Tian et al*.* developed high potential chemo- and magnetic hyperthermia therapeutic platforms based on thermoresponsive copolymers coated magnetic mesoporous silica nanoparticles (MSN)^14^. The nanoparticles showed low cytotoxicity and could be internalized by HeLa cells. The nanoparticles loading with doxorubicin (Dox) exhibited a synergistic effect of chemo- and magnetic hyperthermia therapy, resulting in higher efficacy in killing cancer cells. Similarly, Dox-loaded mesoporous silica nanoparticles functionalized with poly(ethylene glycol)/poly(ε-caprolactone) (PEG/PCL) multiblock copolymer were prepared and used for heat-shock drug delivery system. In response to heat shock stimuli (45 °C), Dox was released from the functionalized MSN particles due to the loosening of the structure of the gatekeeper, PEG/PCL, leading to effective killing of tumor cells^15^.

The development of an efficient encapsulation process, especially for drug carriers, aims to protect the entrapped active agents against light, oxidation, and enzyme degradations^16^. Among drug carriers explored, porous particles have remarkable properties, such as large surface area, high porosity, high uniformity, tunable pore structures, and surface modifiability^17^. Porous cavities can efficiently encapsulate various active compounds with high loading capacity. The particles can be fabricated by both inorganic (mesoporous silica particles)^18^ and organic materials, e.g., poly(lactic acid-*co*-glycolic acid), poly(methyl methacrylate), polylactic acid, and ammonio methacrylate copolymer (Eudragit RS100)^19–21^. Biodegradable Eudragit RS100 was fabricated as porous particles by a double emulsion (W/O/W) solvent evaporation method. A two-step emulsification process was employed for porous particle formation. The water-in-oil (W_1_/O) emulsion was formed by dispersing the inner aqueous phase (W_1_) in organic solvent (O), and emulsifying the primary emulsion in the continuous aqueous phase (W_2_). Zafar et al. reported the fabrication of sponge-like Eudragit RS100 particles *via* a double emulsion solvent evaporation process. The obtained particles possessed porous structures because of the interaction between positively-charged ammonium groups of the polymer and the water molecules. This led to the formation of hydrophilic cavities after solvent evaporation^21^.

Incorporating stimuli-responsive polymers onto porous carriers has been designed to accomplish controlled release formulations by employing the porous particles as hosts and stimuli-responsive polymers as gatekeepers. A gatekeeper can provide the pores’ close/open switchable function when triggered. Various temperature-sensitive gatekeepers have been vastly investigated due to their specific sol–gel phase transition temperatures. Among these, poly(*N*-isopropylacrylamide) (PNIPAM) possesses a lower critical solution temperature (LCST) at around 32 °C in an aqueous solution. Below the LCST, PNIPAM is swollen due to hydrogen bond formation between its amide groups and water molecules. Above LCST, however, the polymer segments become hydrophobic and shrunk^22,23^. When PNIPAM is crosslinked in the form of hydrogels, the critical temperature is called volume phase transition temperature (T_VPT_)^22^. Su et al*.* prepared thermo-responsive composites of hollow silica particles with ordered mesoporous shell coated with PNIPAM layer as a thermo-responsive gate. PNIPAM corona was formed surrounding the hollow silica particles *via* an *in situ* polymerization. The uptake and release of rhodamine B (a model compound) were examined to assess the stimuli-responsiveness of the composite particles. The uptake and release behaviors were observed at 25 °C (< LCST), while the dehydrated hydrophobic polymer layer collapsed over the particle surface at 50 °C (> LCST), leading to a shutoff of the uptake and release^24^. Although the silica-PNIPAM composite particles showed advantages from the hollow mesoporous silica structure and the thermo-responsive polymer gating, a multi-step preparation process is required.

Biodegradation and biocompatibility are essential for materials to use in drug delivery systems. Although PNIPAM has low biodegradability, its application as a structural modifier or co-polymerization with other biodegradable polymers increases their degradation rate^25^. In addition, PNIPAM has been proven a biocompatible polymer. Capella et al*.* synthesized PNIPAM hydrogels *via* free radical polymerization, whose biocompatibility was assessed on mouse preadipocytes cells, human embryonic kidney cells 293, and human lung carcinoma epithelial cells^26^. The cell viability results reflected non-cytotoxicity after 96 h. In a separate study, the biocompatibility of PNIPAM nanoparticles at a high concentration (1000 mg/mL) was examined on two mammalian cell lines, i.e., human keratinocyte (HaCaT) and colon cells (SW480)^27^. The nanoparticles didn’t elicit cytotoxic responses in either HaCaT or SW480 cells, indicating their high biocompatibility. Upon internalization, the particles were engulfed in lysosomes, rendering them effectively harmless. In addition, Eudragit RS100 nanoparticles have been shown to elicit no cytotoxicological responses in SW480 cells, although internalized, suggesting their potential as nanocarriers to the drug delivery system.

In the present work, thermally-sensitive micro- and nanoparticles derived from Eudragit RS100 and PNIPAM (PNIPAM@RS100) are designed to achieve a smart delivery system. PNIPAM is selected as a gatekeeper owing to its extensive biocompatibility and cellular interaction to cover the pores of Eudragit RS100 to block the diffusion of active molecules, especially drugs, with a thermal responsive triggering mechanism. The crosslinked PNIPAM nanogels are polymerized during the formation of Eudragit RS100 porous particles by double emulsion solvent evaporation. The PNIPAM nanogels are then decorated in the cavities of the porous particles to act as gatekeepers. The formation mechanism of the thermoresponsive gates is examined. The effect of chemical initiators on the degree of polymerization of NIPAM in the presence of methylene bis-acrylamide (MBA) crosslinker is investigated at 60 °C for ammonium persulfate (APS) and room temperature for redox initiators (APS/TEMED). The hydrodynamic particle size and electrophoretic mobility were measured to determine the volume phase transition temperature of the crosslinked PNIPAM nanogels. Nile Blue A (NB) is then employed as a model compound. The loading efficiency and releasing behavior of NB-loaded PNIPAM@RS100 are examined to assess their performance in smart, responsive, controlled release applications.

## Experimental

### Materials

Ammonio methacrylate copolymer type BNF (Eudragit RS100), a molecular weight of 32,000 g/mol, was purchased from Evonik, Germany. *N*-isopropylacrylamide (NIPAM) (99% pure) and ammonium persulfate (APS) were purchased from Acros Organics and Bio-Rad, respectively. Dichloromethane (DCM), *N,N,N′,N′*-tetramethylethylenediamine (TEMED, ≥ 99%), *N,N′*-methylenebisacrylamide (MBA, 99%), and Nile Blue A (NB) were purchased from Sigma-Aldrich. Deionized water was used throughout this work. Chemical structures of the main components of thermoresponsive gating particles are shown in Supplemental Fig. S1.

### Preparation of thermoresponsive gating particles (PNIPAM@RS100)

Thermoresponsive gating micro-/nanoparticles (PNIPAM@RS100) were obtained *via* the double emulsion solvent evaporation method. The thermoresponsive components of NIPAM (0.2 g, MBA (0.02 g), APS (0.01 g), and TEMED (10 µL) were dissolved in 2 mL of an internal aqueous phase (W_1_). Eudragit RS100 (1 g) was dissolved in 5 mL dichloromethane organic solvent (O). The aqueous phase was emulsified in the organic phase using an ultrasonicator (Optic ivymen system CY-500) at an amplitude 70% for 2 min. The primary emulsion (W_1_/O) was transferred to 50 mL of an external aqueous phase (W_2_) and homogenized using either Ultra-Turrax (IKA-Werke T25 basic) at 13,500 rpm or ultrasound at amplitude 70% for 10 min. The obtained double emulsion (W_1_/O/W_2_) was evaporated by a rotary evaporator (Nahita model 9300), as shown in Supplemental Fig. S2. PNIPAM@RS100 was purified through centrifugation (Fisherbrand, GT2R Centrifuge) at 5000 rpm for 10 min and washed with DI water to remove residue water-soluble PNIPAM.

The effect of initiator systems on the PNIPAM gatekeeper formation was examined. The redox initiating system involving APS as an initiator and TEMED as a catalyst was employed, as the polymerization of NIPAM can be initiated at room temperature *via* a double emulsion technique. This was compared with APS thermal initiator, which decomposes and generates free radicals at 60 °C for 6 h.

### Characterizations

The particle size and zeta potential of the prepared PNIPAM@RS100 were determined by a microelectrophoresis apparatus (Zetasizer; Malvern, Nano ZS). The samples were diluted and dispersed in 1 mM NaCl before measurements. The particle size and zeta potential of each sample as a function of temperature were measured at a temperature range of 25–55 °C. Each measurement was performed in triplicate. The particle morphology was examined using a transmission electron microscope (TEM; JEOL, JEM-2100 Plus), operated at an accelerating voltage of 200 kV, and a scanning electron microscope (SEM; JEOL, JSM7800F), acquired after gold sputtering at an acceleration voltage of 10 kV. The chemical structures and functional groups of the prepared particles were analyzed by Fourier transform infrared (FTIR) spectrometer in an attenuated total reflection mode (ATR-FTIR; Nicolet iS50, Thermo Scientific).

### The volume phase transition temperature of PNIPAM nanogels

PNIPAM nanogels were synthesized by free-radical polymerization in a surfactant-free process to determine the volume phase transition temperature (T_VPT_). In the reaction flask, 0.2 g of NIPAM monomer and 0.02 g of MBA crosslinking agent were dissolved in 20 mL distilled water by stirring with a magnetic bar for 30 min. The temperature was then raised to 70 °C. Subsequently, 10 mg APS was added to the solution to initiate the reaction. The polymerization was carried out at 70 °C for 4 h. The solution became turbid after mixing. The unreacted monomers were separated using a centrifuge filter (0.1 µm pore size). T_VPT_ was investigated by measuring the hydrodynamic particle size as a function of temperature from 25 to 55 °C, after being diluted in 1 mM NaCl medium. The electrophoretic mobility (*µ*) of the particles was measured as a function of temperature using a Zetasizer. The swelling ratio of the nanogels is defined as the volume of swollen PNIPAM nanogels (equilibrium swelling at 25 °C) divided by that of the collapsed state (at 55 °C) according to the following equation^28^.

1 $$\text{Swelling ratio } = {\left(\frac{{\text{D}}_{\text{s}}}{{\text{D}}_{\text{c}}}\right)}^{3} \times 100$$

where D_s_ and D_c_ are average hydrodynamic diameters of nanogels in the swollen and collapsed states, respectively.

### Embedding of PNIPAM gatekeepers into porous particles

The content of the PNIPAM gatekeepers in the porous particles was determined by calculating the percentage of NIPAM conversion. The unreacted NIPAM monomer in the emulsion was collected by centrifugation. The supernatant was dried, whose chemical structures were examined by FTIR spectroscopy. The results confirm that the compounds suspended in the supernatant are NIPAM monomer residues, as shown in Supplemental Fig. S3. Finally, the mass of the polymerized PNIPAM, and % conversion were evaluated by using Eqs. (2) and (3), respectively

2 $${\text{M}}_{i} = {\text{M}}_{wps} + {\text{M}}_{p}$$

3 $$\% \text{Conversion} = \frac{{\text{M}}_{p}}{{\text{M}}_{i}} \times 100$$

where M_i_ is the mass of the initial monomer, M_wps_ is the mass of dried unreacted monomer in the supernatant, and M_p_ is the mass of polymerized PNIPAM.

### Encapsulation efficiency and loading capacity

NB is employed as a model compound in the encapsulation process. NB (1 mg/mL) was added to the primary aqueous solution (W_1_), before PNIPAM@RS100 formation. The encapsulated particles were centrifuged to separate the particles and the supernatant. The collected supernatant was analyzed using a UV–Vis spectrophotometer (UV-1800, Shimadzu) at 635 nm. All samples were measured in triplicate. The encapsulation efficiency (%EE) and loading capacity (%LC) were calculated using Eqs. (4) and (5), respectively.

4 $$\% \text{EE } = \frac{\text{Initial amount of NB} - \text{Amount of NB in supernatant}}{\text{Initial amount of NB}} \times 100$$

5 $$\% \text{LC } = \frac{\text{Initial amount of NB} - \text{Amount of NB in supernatant}}{\text{Weight of nanoparticles}} \times 100$$

### Controlled release mechanism

In the study of NB release behavior, 0.2 mg of NB-loaded PNIPAM@RS100 was suspended in distilled water. A drop (5 µL) of 0.1 M hydrochloric acid (HCl) was added into the releasing medium to aid the observation of NB color. The real-time NB release was monitored continuously using a UV–Vis spectrophotometer (UV-1800, Shimadzu) at 635 nm for 5 h, at 25.0 ± 0.5 and 37.0 ± 0.5 °C. Finally, the cumulative release was determined from a standard calibration curve (Supplemental Fig. S4).

The switchable close/open gating properties of the materials were evaluated by incubating at 4 and 40 °C. The crosslinked PNIPAM gatekeepers were turned off at a low temperature (4 °C) for 5 min and turned on for 5 min allowing NB to release above the T_VPT_ (40 °C). This process was repeated for four cycles. The released NB content was calculated.

## Results and discussion

### Design and formation of thermoresponsive gating particles (PNIPAM@RS100)

**Figure 1:**
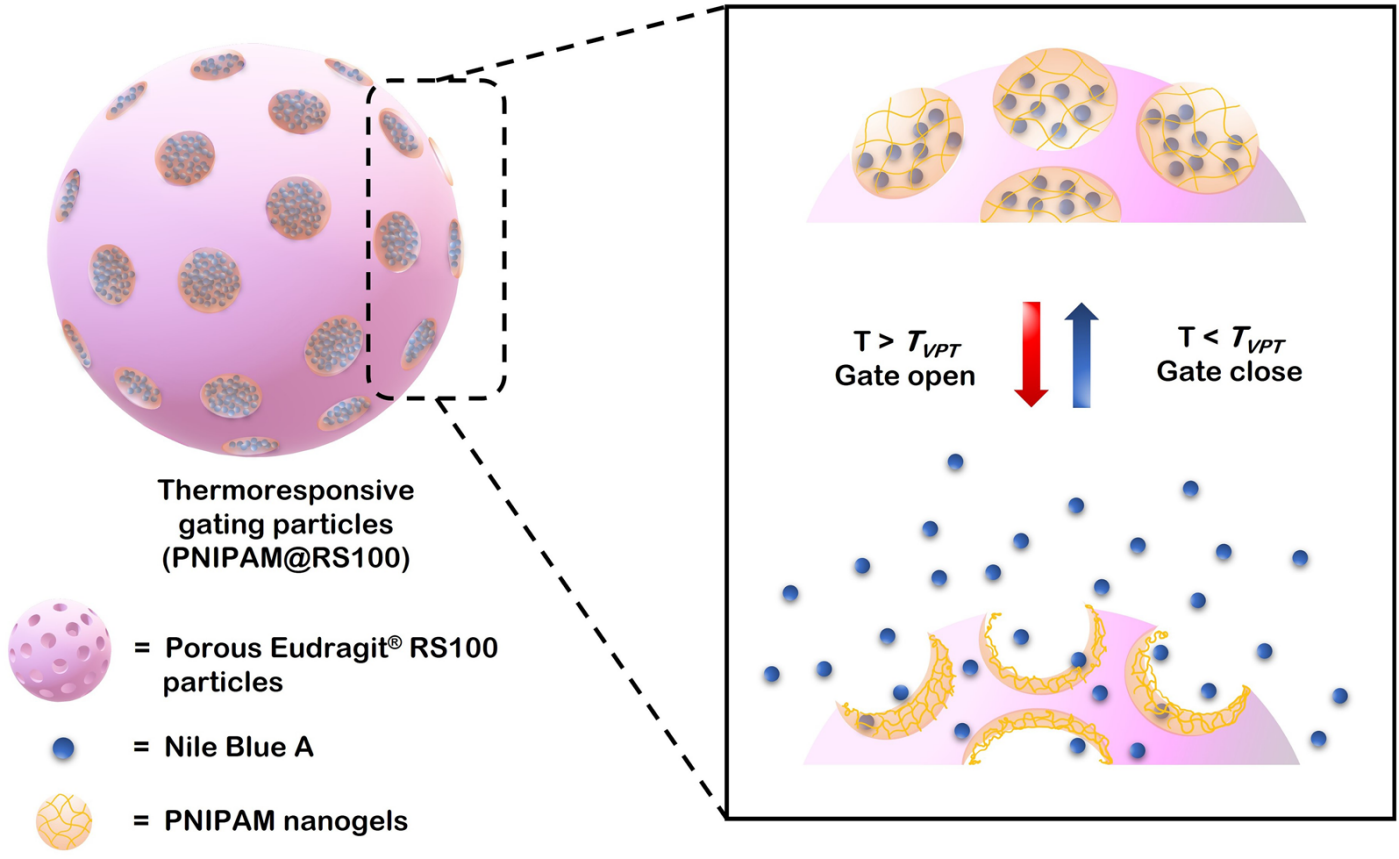
Design of thermoresponsive gating particles and the controlled release mechanism of thermally-sensitive PNIPAM gatekeepers.

A schematic representation of the thermoresponsive PNIPAM gating system for controlled delivery is shown in Fig. 1. The porous domain structure of Eudragit RS100 was successfully prepared without any stabilizer, as reported in the literature^21^. The PNIPAM gates were produced during the PNIPAM@RS100 formation *via* a double emulsion solvent evaporation. In the first step, water-soluble compounds of thermoresponsive gates and NB were dissolved in the aqueous inner W_1_ phase, then dispersed into the organic phase. The W_1_/O nanodroplets were converted into PNIPAM nanogels after polymerization, and NB was encapsulated inside the nanogels. A double emulsion (W/O/W) was formed by dispersing the primary emulsion in a secondary water phase (W_2_). Finally, the organic solvent was evaporated, resulting in the formation of PNIPAM@RS100.

The NB model compound was encapsulated within the PNIPAM nanogels in the porous Eudragit RS100 particles. Upon increasing the temperature above T_VPT_, a collapse in the PNIPAM network structure leads to an opening of the pores and a release of the model compound.

### Structure and properties of PNIPAM gatekeepers

**Figure 2:**
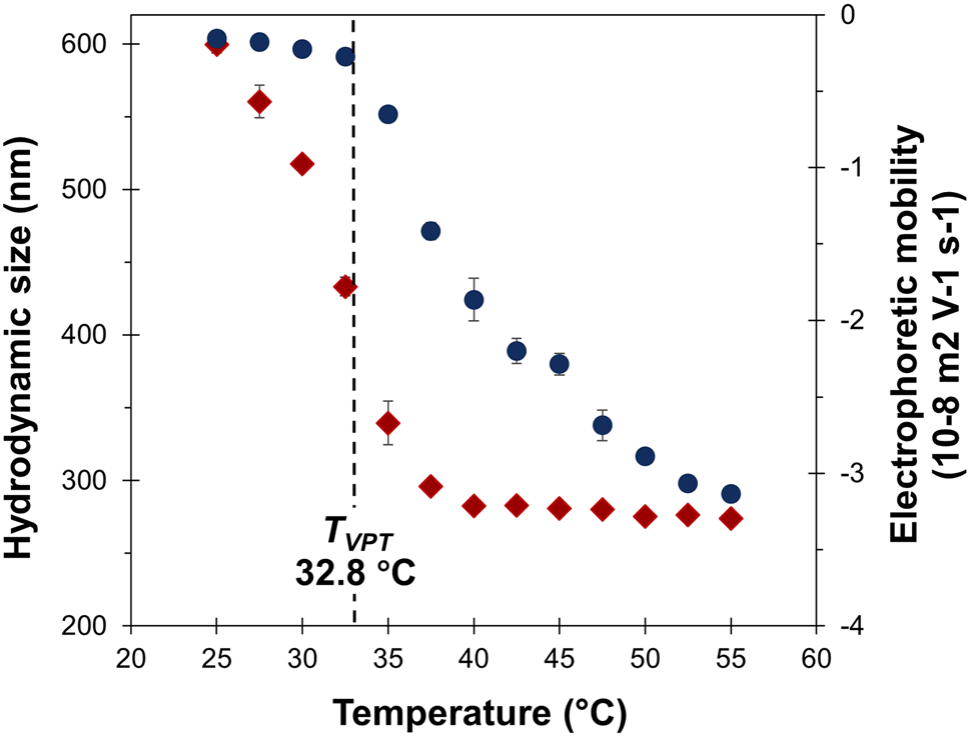
The effect of temperature on hydrodynamic particle size (red rhombus) and electrophoretic mobility (dark blue circle) of the crosslinked thermally-sensitive nanogels.

PNIPAM nanogels were fabricated by radical polymerization to determine T_VPT_ of the thermally-sensitive gatekeepers. The results on average particle sizes of the PNIPAM nanogels as a function of temperature from 25 to 55 °C are summarized in Fig. 2. All samples showed monodisperse size distribution with a polydispersity index (PDI) < 0.1. This indicates that the hydrodynamic diameter tends to decrease with increasing temperature. T_VPT_ was calculated from the first derivative of the first transition step (start to collapse, around 27 °C), and the second transition at around 40 °C, using a polynomial fitting^29,30^. Therefore, the observed T_VPT_ is at 32.8 °C. The variation in the temperature plays a key role in the hydrodynamic size of the crosslinked nanogels. The swelling and shrinking behaviors are due to the breaking of hydrogen bonds between PNIPAM networks and water molecules during the temperature escalation, resulting in a decrease in the particle’s hydrodynamic size^31^.

The swelling ratio is defined as a ratio between the volumes of the nanogels in the swollen and the collapse states. At 1% of MBA crosslinker content, the swelling ratio of the prepared nanogels was 10.5. The swelling ratio is related to the crosslink density of the hydrogels. The increase in the crosslinker concentration leads to higher crosslink density. However, the water absorption capacity is reduced^28,32^. In addition, T_VPT_ of the thermally-sensitive particles is dependent on the monomer types and the monomer/crosslinker ratio. Varga et al*.*^33^ reported that an increase in the crosslink density reduced the swelling behavior of PNIPAM microgel particles and shifted their T_VPT_ value, as a result of crosslinker content differences. The sol–gel transition was observed upon temperature changes. The nanogels form a sol phase at room temperature or below T_VPT_, while at temperatures higher than T_VPT,_ these become hydrophobic or a gel phase due to hydrogen bonding disruption, leading to a turbid solution^29,34^.

The electrophoretic behavior reflects the motion of thermosensitive particles under external electric and friction forces due to their charge density^35^. Figure 2 also demonstrates the electrophoretic mobility of PNIPAM particles as a function of temperature. The negative electrophoretic mobility values indicated that these nanogels were negatively charged inherited from sulfate groups of the APS anionic initiator. The results on the effect of cationic and anionic initiators on the surface charge density and electrophoretic mobility of the particles agree with those previously reported^30^. Below T_VPT_ (at 32.8 °C), the electrophoretic mobility of nanogels was near zero with negatively charged, because of the low surface charge density of the swollen particles. The change in electrophoretic mobility was observed when the temperature was increased above T_VPT_. The value also increased with the decrease of particle diameter, as the charges located on the hydrodynamic surface were closer to each other, leading to an increase in the surface charge density. This phenomenon is likely because the shrinkage of particles induces the reorganization of the charged groups, and enhances the surface charge density^30,36^. The relationship between temperature and electrophoretic mobility can be explained by the Helmholtz-Smoluchowski’s equation^37,38^, in which the electrophoretic mobility (*µ*_e_) is correlated with the surface charge density (σ) and hydrodynamic particle size (2r_h_), as follows:

6 $${\mu }_{e} \approx \frac{Ne}{4\pi {\eta \kappa {r}_{h}}^{2}}$$

where N is the number of charge groups, e is the electronic charge, η is the viscosity of the medium, and κ is the reciprocal Debye length.

### Structure and properties of thermoresponsive gating particles (PNIPAM@RS100)

**Figure 3:**
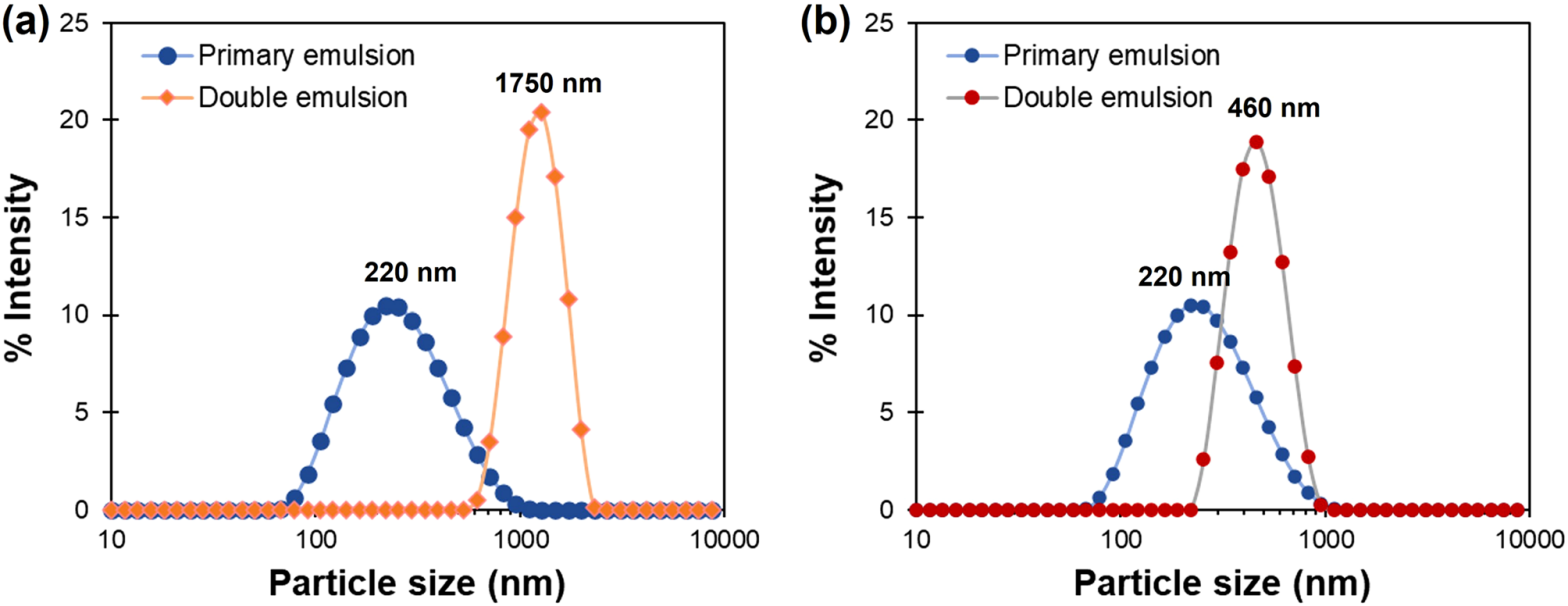
Particle size distribution of a primary W_1_/O emulsion and a double W_1_/O/W_2_ emulsion of Eudragit RS100 particles, prepared by using (**a**) ultra turrax (UT) and (**b**) ultrasound (US) in the secondary step.

The formation of thermoresponsive gating porous particles (PNIPAM@RS100) was obtained by the double emulsion (W/O/W) solvent evaporation technique. The double emulsion was prepared *via* a two-step emulsification process, in which a primary (W_1_/O) emulsion was employed to form PNIPAM gatekeeper nanogels. These were then dispersed in Eudragit RS100 solution to formulate a double emulsion (W_1_/O/W_2_). The primary emulsion as the oil phase was transferred to another aqueous phase under a high-speed homogenizer ultra turrax (UT) or a high-energy ultrasound (US). The results on the average size and size distribution are summarized in Fig. 3. The nanodroplets in the primary emulsion showed hydrodynamic diameters between 90 and 950 nm with a PDI value of 0.2, reflecting monodispersed emulsion. The high-energy sonication was employed to produce uniform and smaller droplets in the primary emulsion relative to the larger counterparts obtained in the double emulsion process. Nevertheless, the small droplet size does not affect the final particle size in the secondary emulsification^21,39^. The energy required to form secondary emulsion was provided to the system by UT and US to avoid aggregation and enhance particle dispersion.

The utilization of UT produced microparticles with an average particle size of 1750 nm and a PDI value of 0.4 (Fig. 3a). In contrast, when US was applied in the secondary emulsion process, nanoparticles with an average size of 460 nm and narrow size distribution (PDI value of 0.2) were produced, as shown in Fig. 3b. Although both nano- and microparticles showed high colloidal stability at ambient temperature, the colloidal microparticles fabricated by UT precipitated after 7 days, likely due to the gravitational force, as shown in Supplementary Fig. S5. However, the microparticle sediment can be easily re-dispersed upon shaking or agitation. In contrast, the smaller nanoparticles are well-dispersed without sedimentation or aggregation^21,40,41^.

**Figure 4:**
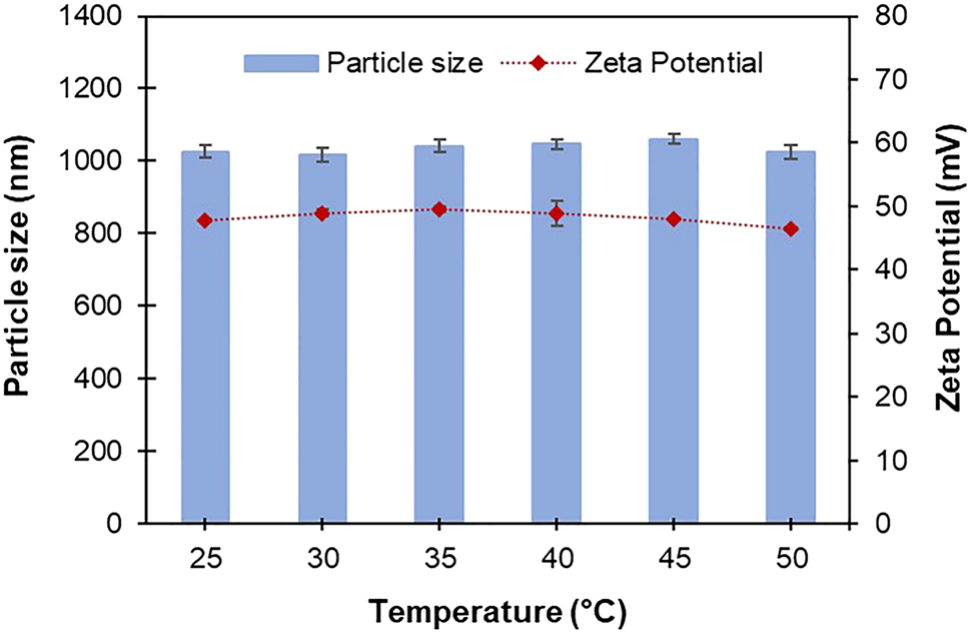
Average size and zeta potential of PNIPAM@RS100 measured at different temperatures.

The obtained emulsions are stable without any stabilizer, which was confirmed by a high zeta potential value of >  + 40 mV, because of the positively-charged nature of Eudragit RS100. The incorporation of PNIPAM didn’t significantly affect the zeta potential value of the particles. The zeta potential values of neat Eudragit RS100 and PNIPMA nanogels were observed at + 48 mV and − 15 mV, respectively. The change in temperatures also did not affect the particle size and zeta potential of PNIPAM@RS100, as shown in Fig. 4. This is because the matrix domain of these particles is derived from porous Eudragit RS100, which is not sensitive to temperature changes. It is noted that the cationic macromolecule Eudragit RS100 could interact with anionic components (*N*-acetyl-neuraminic acid) of glycoprotein on the surface of cells. However, the materials are non-toxic to human cells. Lopedota et al*.* examined the *in vitro* cytotoxicity of Eudragit RS100 (zeta potential of + 40.5 mV) with a concentration in the range of 0–500 µg/mL on human epithelial cells (HaCaT) and murine monocyte-macrophage cells (RAW 264.7)^42^. The authors reported no detectable cytotoxicity after 48 and 72 h of incubation.

The polymerization of thermally-sensitive PNIPAM gatekeepers takes place during the PNIPAM@RS100 preparation step by a free radical polymerization. The effect of initiators on the degree of polymerization of NIPAM was investigated at 60 °C for a thermal initiator (APS) (referred to PNIPAM@RS100-APS), and at room temperature for redox initiators (referred to PNIPAM@RS100-APS/TEMED). The redox initiator system was used to initiate the polymerization below the decomposition temperature of APS^43^. A small amount of TEMED catalyst was added into the inner aqueous phase (W_1_). The catalyst can help enhance the polymerization rate by decreasing the activation energy (E_a_) of the reaction at room temperature^44^. The redox initiator system was employed to polymerize PNIPAM networks at room temperature. The APS/TEMED redox pair (ratio 1:1) generated the primary initiating radicals (HSO_3_O^•^ and (CH_3_)_2_NCH_2_CH_2_N^•^(CH_3_)_2_), and hydroxyl free radicals (OH^•^) via a charge transfer complex and cyclic transition state in water medium^44,45^. These reactive species initiated the NIPAM monomers and MBA crosslinker. A chain propagation of NIPAM then creates chain entanglements and covalent crosslinking of the PNIPAM structures (Supplemental Fig. S6). With the addition of TEMED, the percent conversions of NIPAM monomer to polymer were enhanced, while the particle diameter, polydispersity index, and zeta potential of both emulsions are in the same range, as summarized in Table 1.

**Table 1:** Average particle size, polydispersity index (PDI), zeta potential, and the conversion percentage of NIPAM monomer during the preparation of PNIPAM@RS100 particles.

Sample	Particle size (nm)	PDI	Zeta potential (mV)	Conversion (%)
UT-APS	1679 ± 81	0.47	48.3 ± 0.4	66.7 ± 0.5
UT-APS/TEMED	1729 ± 88	0.65	48.8 ± 0.5	77.3 ± 1.9
US-APS	415 ± 3	0.22	47.7 ± 0.8	69.9 ± 1.9
US-APS/TEMED	430 ± 7	0.15	47.8 ± 0.2	84.0 ± 2.2

Mean ± SD, n = 3, SD: standard deviation.

**Figure 5:**
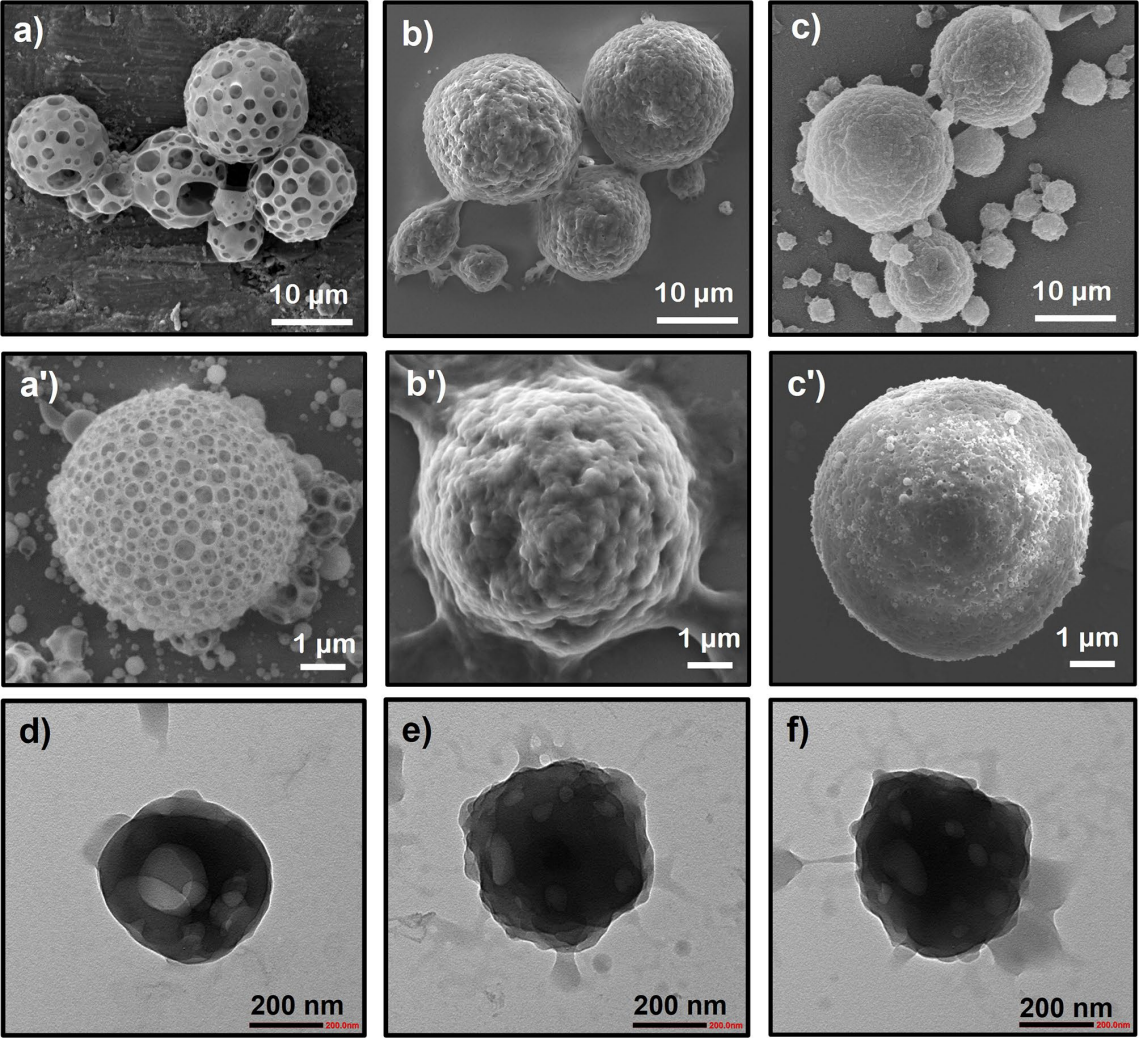
SEM images of Eudragit RS100 porous particles and PNIPAM@RS100 prepared by ultra turrax; (**a**,**a′**) UT-RS100, (**b**,**b′**) UT-APS, (**c**,**c′**) UT-APS/TEMED, and TEM images of nanoparticles prepared by ultrasonication; (**d**) US-RS100, (**e**) US-APS, (**f**) US-APS/TEMED. The images were taken at 5kX (**a**–**c**), 10kX (**a′**–**c′**) and 25kX (**d**–**f**) magnifications.

**Figure 6:**
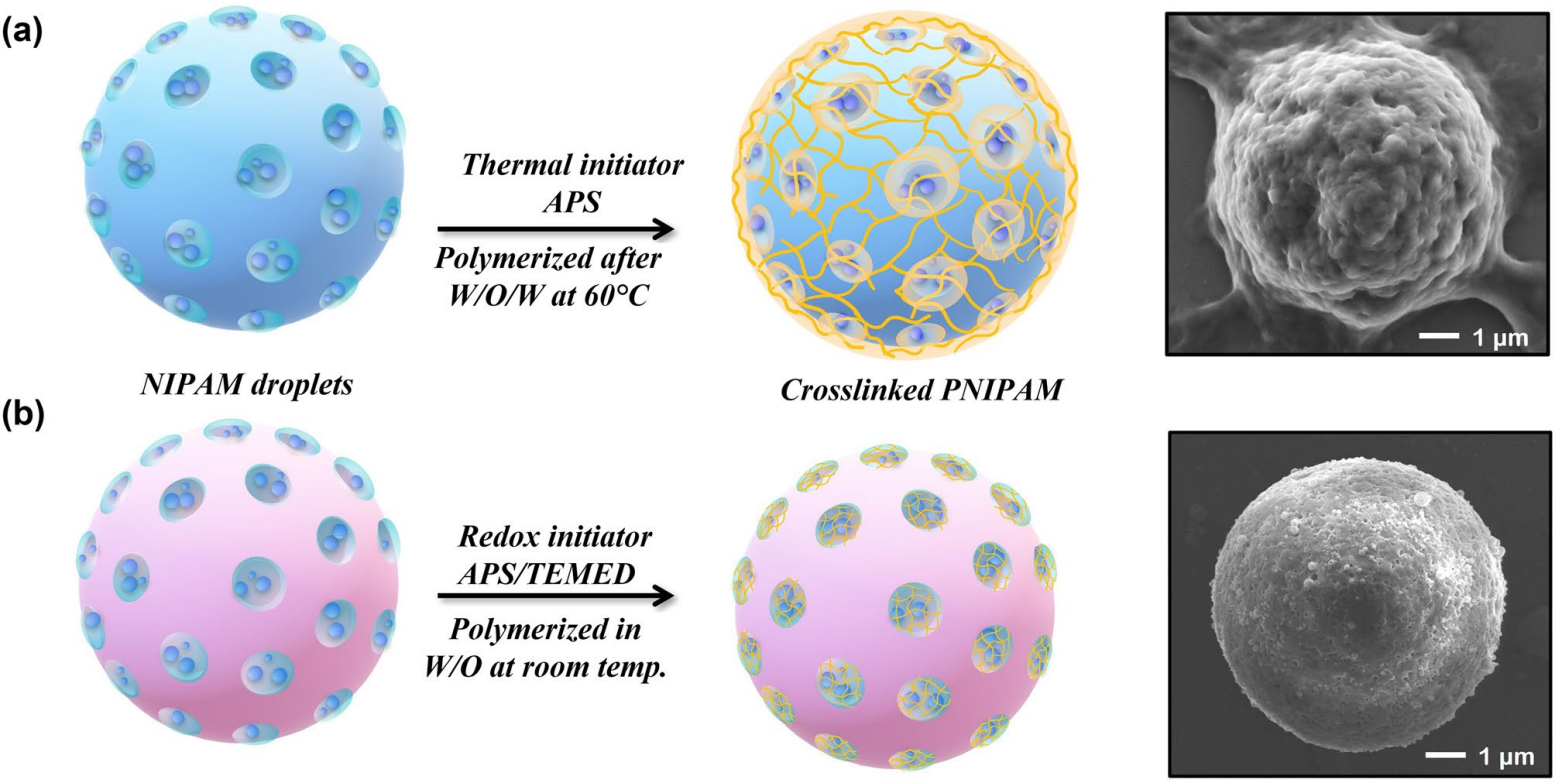
Schematic of the formation mechanisms of PNIPAM gatekeepers on porous Eudragit particles by using different initiators; (**a**) thermal and (**b**) redox initiators.

The materials are designed to contain 20% w/w of PNIPAM in the Eudragit RS100 matrix. The feed concentration of NIPAM monomer in water was fixed at 1 wt/v% for all samples^46^. Under the redox system, high concentrations of PNIPAM were incorporated in the pores of Eudragit RS100 (15.5 and 16.8%), while 13.3 and 14.0% were obtained for the thermal system counterpart, using UT and US methods, respectively. The resulting PNIPAM contents may be correlated with the porous particle formation and hence their thermo-responsive properties. To confirm this hypothesis, the morphology of the porous particles and thermoresponsive particles prepared by thermal and redox initiators are compared in Fig. 5. Spherical particles with large cavities and smooth surfaces were obtained in the UT system. After the polymerization, some cavities were closed or covered with a rough surface, likely derived from crosslinked PNIPAM domains. In contrast, PNIPAM@RS100-APS showed growing polymer chains enveloping the particles, as PNIPAM was polymerized after the particle formation. This is because the hydrophilic chains tend to move out to the aqueous medium, as they are allowed extensive time for polymerization. In the redox initiating system (PNIPAM@RS100-APS/TEMED), however, the PNIPAM nanogels were polymerized during the W/O formation in the first stage. The proposed formation mechanism of PNIPAM gatekeepers is shown in Fig. 6. In the US process, the high vibrational energy induced cavitation in the double emulsion process. TEM images show that neat Eudragit particles have a smooth surface and a large cavity inside the particles. The functionalized PNIPAM particles, however, exhibited rough surfaces, as the PNIPAM chains were present both inside and at the surface of the nanoparticles.

**Figure 7:**
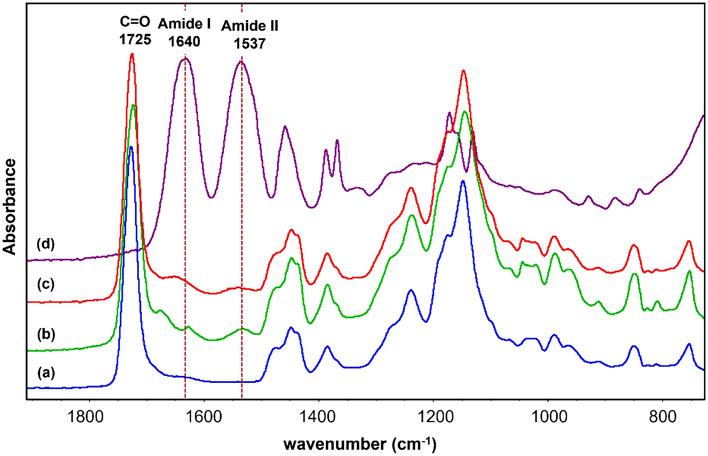
ATR-FTIR spectra of (**a**) neat Eudragit RS100 porous particles, (**b**) PNIPAM@RS100-APS, (**c**) PNIPAM@RS100-APS/TEMED, and (**d**) PNIPAM nanogels.

The coexistence of Eudragit RS100 and PNIPAM gates is confirmed by ATR-FTIR spectroscopy, as shown in Fig. 7. The spectrum of neat porous particles shows the characteristic band at 1725 cm^−1^, corresponding to the stretching mode of C=O of Eudragit RS100^47^. PNIPAM nanogels show bands at 1640 and 1537 cm^−1^, corresponding to the amide I (C=O stretching) and amide II (N–H bending) modes, respectively^48^. The corresponding spectra of thermoresponsive particles PNIPAM@RS100 were almost identical to that of neat Eudragit RS100 particles. The appearance of the amide I and II bands confirms the formation of PNIPAM gatekeepers on the porous particles from the conversion of the NIPAM monomer.

### Encapsulation and in vitro release of Nile Blue A (NB)

**Figure 8:**
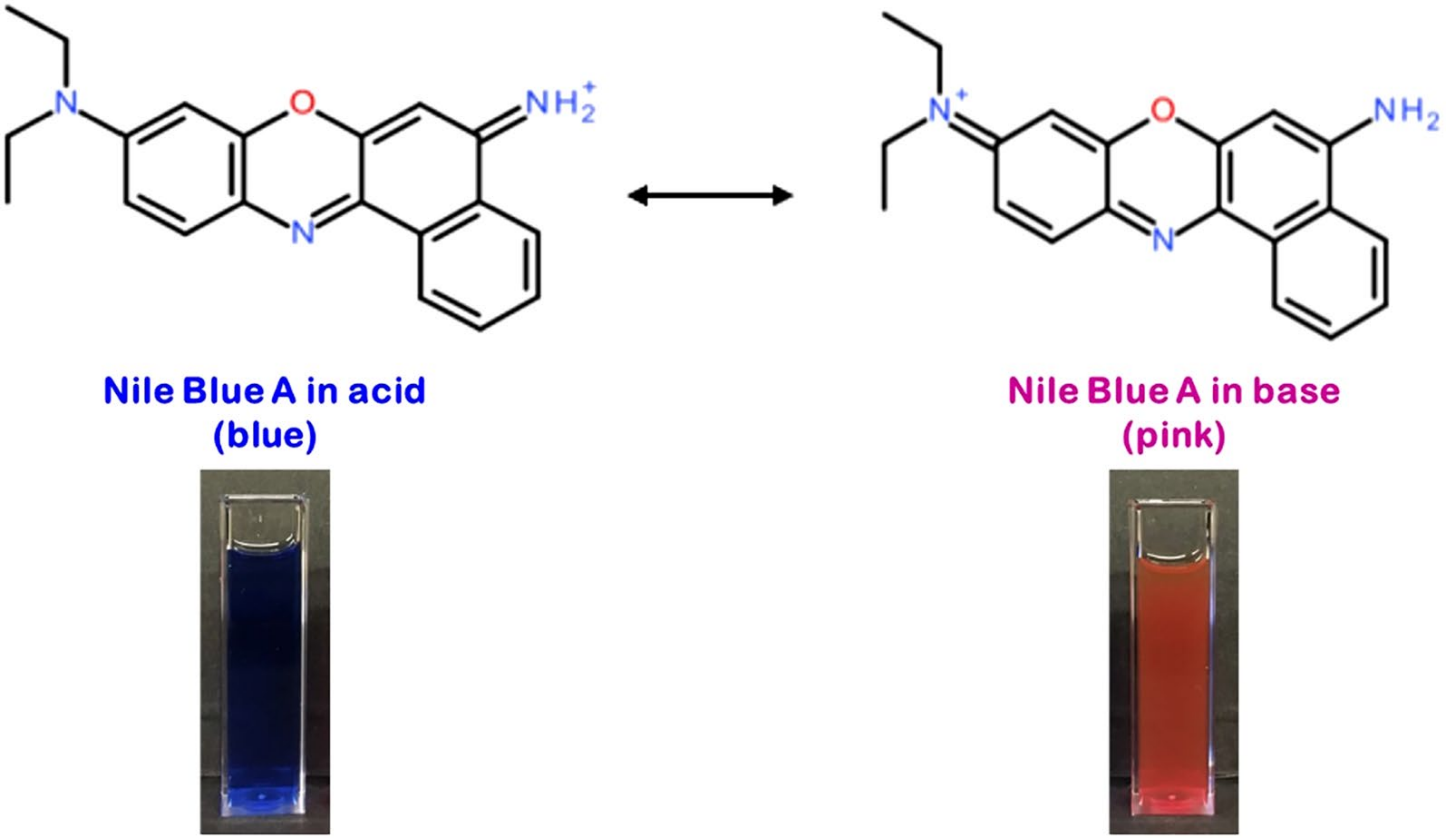
Reversible protonation and deprotonation states of Nile Blue A.

To investigate the encapsulation efficiency and release behavior of PNIPAM@RS100-APS/TEMED, a fluorescent dye NB is chosen as a model compound. The dye also serves as a pH-sensitive probe, whose colorimetric reversibly changes from blue to pink, depending on acidic or basic (pH > 7.6) environments by the protonation/deprotonation of its amino groups and the rearrangements of its conjugate system^49^. The cationic charge transfer structures of NB are demonstrated in Fig. 8.

**Figure 9:**
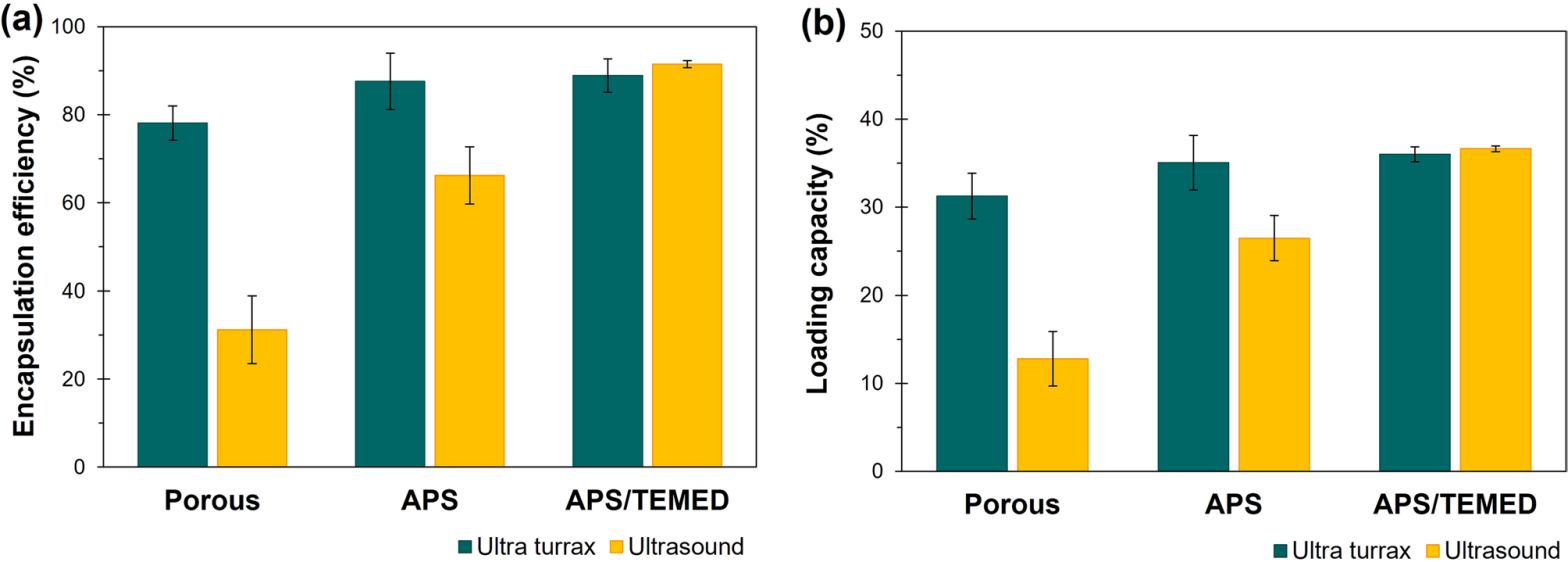
Comparative study of (**a**) encapsulation efficiency and (**b**) loading capacity of Nile blue A-loaded particles prepared by ultra turrax (UT) and ultrasound (US) in the second step.

The encapsulation of the NB model in PNIPAM@RS100 particles was conducted by direct encapsulation during the particle’s formation step. NB was uniformly distributed and covered with crosslinked PNIPAM gatekeepers. The encapsulation efficiency (%EE) and loading capacity (%LC) of NB were calculated according to Eqs. (4) and (5), as summarized in Fig. 9. The encapsulation efficiency is dependent on the presence of the PNIPAM gating systems, especially those prepared by US. Given the same NB feed content of 0.2 mg for all samples, the nanoparticles prepared by US showed 92%EE and 36%LC for US-APS/TEMED, 66%EE and 27%LC for US-APS, and 32%EE and 13%LC for neat porous particles. In contrast, the microparticles prepared by UT showed 89%EE and 36%LC for UT-APS/TEMED, 88%EE and 35%LC for UT-APS, and 78%EE and 32%LC for neat porous particles. This implies that the presence of PNIPAM gatekeepers is essential to effectively entrap NB inside PNIPAM@RS100. In the microparticle system, high encapsulation efficiency was observed due to the large pore volume that could encapsulate a high amount of NB in their porous structure. In addition, the type of initiators used in the PNIPAM polymerization plays a key role in the encapsulation efficiency. The results from the redox-initiated system of PNIPAM gatekeeper’s formation strongly support an assumption that the addition of TEMED helps to improve the polymerization rate of the crosslinked PNIPAM, resulting in the highest effective gatekeepers in the entrapment of NB.

**Figure 10:**
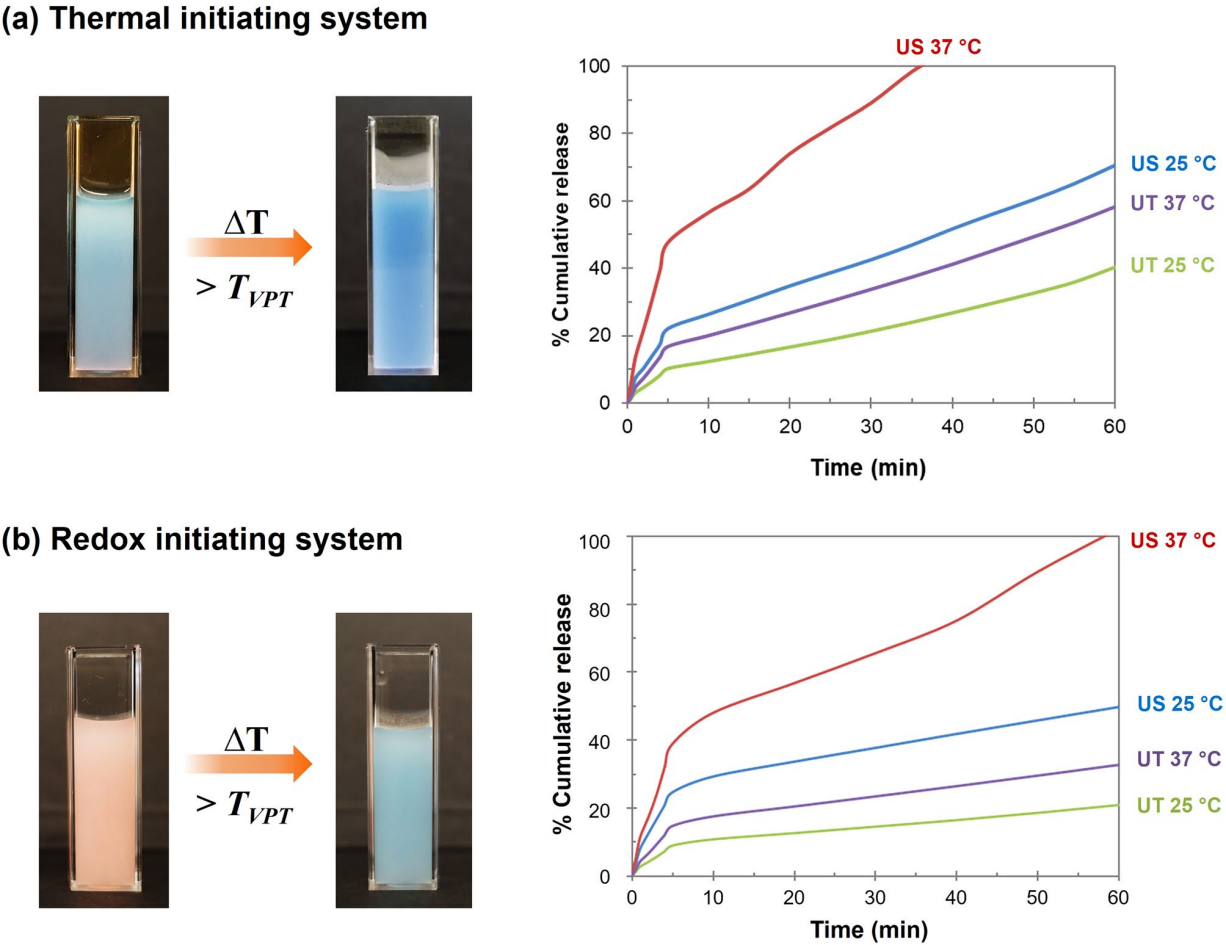
Thermoresponsive behavior of PNIPAM@RS100; visual observation of color change of emulsions and cumulative release of Nile Blue A at room temperature (25 °C) and the body temperature (37 °C) in DI water. The PNIPAM gating particles were prepared by (**a**) thermal and (**b**) redox initiating systems.

The temperature-sensitive gating behavior of the biodegradable porous particles was studied by monitoring the release of the NB model. In the thermal-initiating system, NB encapsulated in PNIPAM@RS100-APS emulsion is blue due to the acidic environment (pH of 3.4). In contrast, a pink emulsion was observed in the redox-initiating counterpart (PNIPAM@RS100-APS/TEMED) because of the contact between NB and basic TEMED (pH of 8.9). From visual inspection, the NB-loaded colloidal particles changed from pink to blue when the temperature was increased above T_VPT_, as the entrapped NB (pink form) was released to a neutral medium (blue form). To evaluate the performance of the crosslinked PNIPAM gatekeepers, the NB release profiles from PNIPAM@RS100 carriers were examined at room temperature (25 °C, below T_VPT_) and the human body temperature (37 °C, above T_VPT_). A spectrometer was employed to monitor the kinetic release of NB as a function of time. The cumulative release of NB from thermoresponsive particles measured at 25 and 37 °C is summarized in Fig. 10. The results indicate an initial burst release in the early state (5 min), followed by a diffusive release. The zero-order kinetic release indicated a drug diffusion-controlled mechanism through PNIPAM gatekeepers and the particle cavities. The released NB at room temperature was around 50%. By increasing the temperature above T_VPT_, the cumulative release rate increased as the crosslinked PNIPAM collapsed, causing the opening of gatekeepers. The loaded NB in US-PNIPAM@RS100 was released at a higher rate and reached 100% within one h. For microparticles with larger pore sizes, UT-PNIPAM@RS100 showed a prolonged release mechanism. The results agree with Chen et al*.* on the effect of particle size on the in vitro release of PLGA particles^11^. The size-fractionated particles showed different drug release rates, i.e., small size particles exhibited a rapid diffusion and a complete release within one week, while larger microspheres showed slower release for more than three months. This suggested that the diffusion rate of the entrapped drug trapped inside the cavity is correlated with the diffusion path lengths between the interior cavity and the wall-cavity interface.

After the complete release experiments at 25 and 37 °C, the morphology of all NB-encapsulated particles remained unchanged, as evidenced in Supplementary Fig. S7. This is due to the high thermal stability of their Eudragit RS100 matrix. Given that Eudragit RS100 has a glass transition temperature (T_g_) at 57 °C, the material remains in a glassy state throughout the temperature range. This confirms that the crosslinked PNIPAM gatekeepers play a key role in controlling the release of the model compound when triggered by temperature changes.

**Figure 11:**
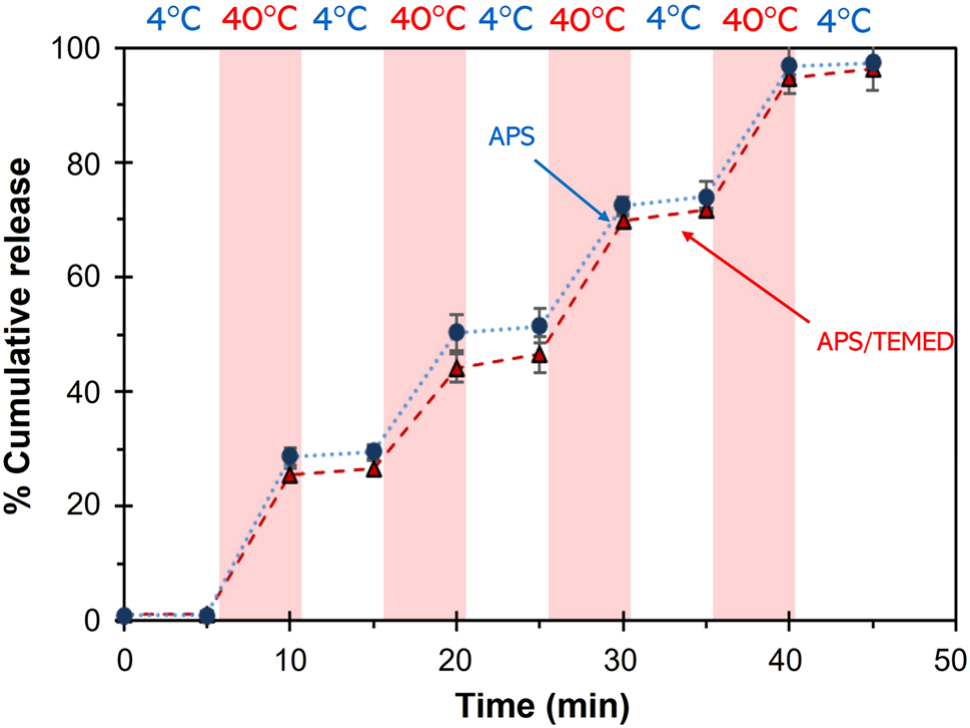
Switchable release profiles of US-PNIPAM@RS100.

In medical applications, especially hyperthermia cancer treatments, a precise and rapid drug release is essential when the materials are triggered, rather than a steady release, because a high dose is required to kill the tumor cells^50^. As PNIPAM has T_VPT_ at 32.8 °C, the temperatures in the ranges enveloped at this point can be used to stimulate its gating behavior. To demonstrate a precise control of the gatekeeping mechanism, a temperature pair with a sufficiently large difference within the normal body temperature range is chosen at 4 and 40 °C. The nanoparticles prepared by the US method were examined, as shown in Fig. 11. The release behavior is also demonstrated as video supplemental data. These phenomena are related to the pore structure of the particles and the responsive behavior of the polymeric gatekeepers. When the temperature was increased above T_VPT_ (40 °C), as “turn on”, the collapsed PNIPAM network allowed NB to diffuse out (*ca.* 25% during a period of 5 min). The gatekeeper was then “closed” by cooling down to 4 °C. A similar amount of NB was further released when the materials were reheated to 40 °C in the following cycles. Although a trace amount of NB was released due to delayed movements of the closing gates, the constant release rate reflects a well-defined release mechanism. Almost all NB was released after four cycles of experiments, indicating complete removal of the encapsulated compound.

## Conclusions

Thermoresponsive PNIPAM network has been demonstrated as an effective gatekeeper for biodegradable porous particles of Eudragit RS100 to entrap and release a model compound (NB). The porous particles functionalized with PNIPAM gatekeepers were synthesized by a facile in situ formation *via* a double emulsion solvent evaporation. The reaction time is much shorter than other reported multi-step processes, consisting of several particle formation steps and functionalization with stimuli-responsive agents. The employment of a redox-initiated system (APS/TEMED) in the fabrication of the nanoparticles improved the gatekeeper formation and encapsulation efficiency (92%). The NB-loaded PNIPAM@RS100-APS/TEMED particles were selected to demonstrate a switchable “close-open” response mechanism of the temperature-sensitive gates. Colorimetric change (pink to blue) of the emulsion of NB-loaded particles reflected the collapsed gates and drug diffusion. The gatekeeper’s switchable open/close mechanisms can be accurately controlled to release the loaded compound at a constant rate until completion. The results indicate that the PNIPAM@RS100 thermoresponsive system is a promising candidate for controlled drug delivery materials at the human body temperature, with a precise and easy triggering mechanism.

## Supplementary Information

Supplementary Figures.

Supplementary Video 1.
