# Supplementary material for: Biodegradable porous micro/nanoparticles with thermoresponsive gatekeepers for effective loading and precise delivery of active compounds at the body temperature
Source: Sci Rep. 2022 Jun 28;12:10906. doi: 10.1038/s41598-022-15069-x (PMC9240026; doi:10.1038/s41598-022-15069-x)
Supplement: Supplementary file 1 — Supplementary Figures. [file 41598_2022_15069_MOESM1_ESM.docx]

# **Biodegradable porous micro/nanoparticles with thermoresponsive gatekeepers for effective loading and precise delivery of active compounds at the body temperature**

Kamonchanok Thananukul^1, 2^, Chariya Kaewsaneha^1^, Pakorn Opaprakasit^1^*, Nadia Zine^2^, Abdelhamid Elaissari^2^*

^1^ School of Bio-Chemical Engineering and Technology, Sirindhorn International Institute of Technology (SIIT), Thammasat University, Pathum Thani 12121, Thailand

^2^ Univ Lyon, University Claude Bernard Lyon-1, CNRS, ISA-UMR 5280, 69622 Villeurbanne, France

*Corresponding authors: pakorn@siit.tu.ac.th, abdelhamid.elaissari@univ-lyon1.fr

Supporting Information


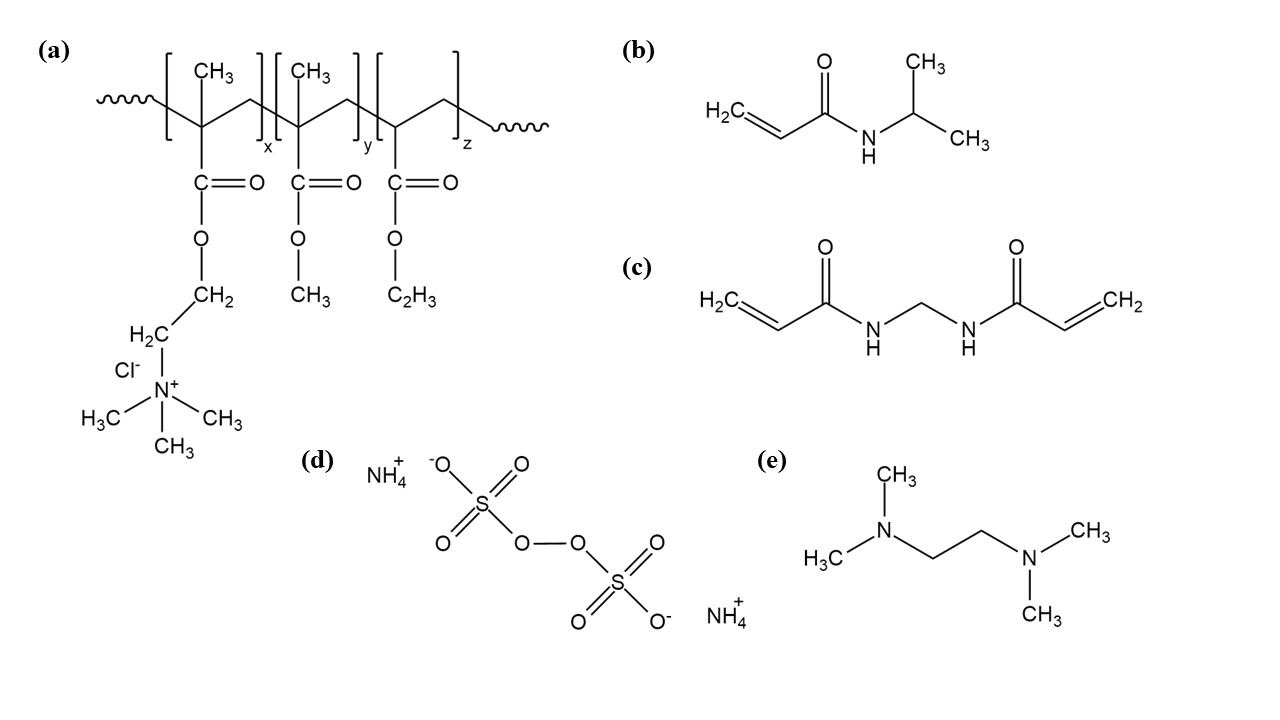


**Figure S1** Chemical structures of (a) Eudragit RS100, (b) NIPAM, (c) MBA, (d) APS, and (e) TEMED.


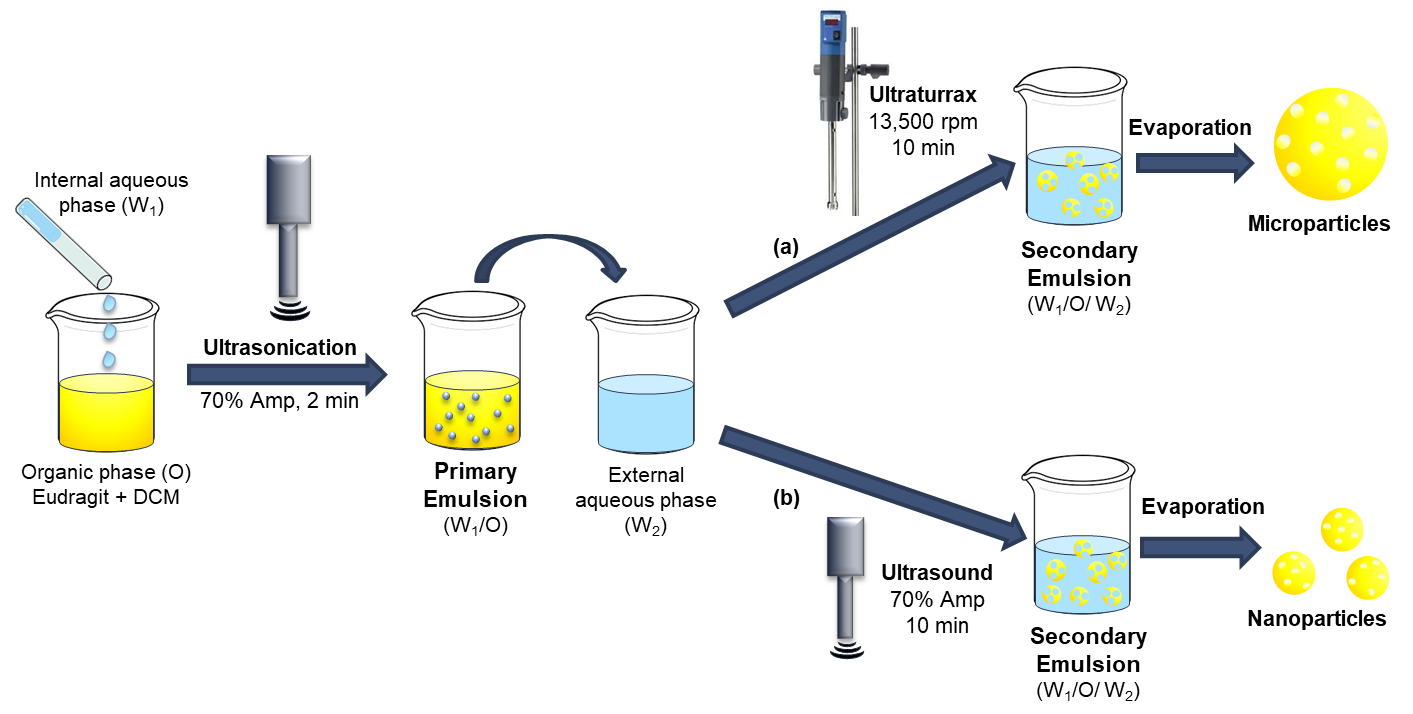


**Figure S2** Schematic illustration of the PNIPAM@RS100 formation by double-emulsion solvent evaporation technique.

ATR-FTIR spectra of the monomer (spectrum a) and unreacted NIPAM in the supernatant (spectrum b) showed typical absorption bands at 1656 and 1620 cm^-1^, corresponding to the C=O stretching mode of amide I. The band at 1546 cm^-1^ is assigned to the N-H bending of amide II. In the polymerized PNIPAM nanogels (spectrum c), the amide I band is broader as it overlaps with the O-H vibration of water molecules. The C=O groups and bound water molecules interact through hydrogen bonding, reflected by the appearance of the amide I band of PNIPAM at 1640 cm^-1^. The characteristic modes at 1386 and 1369 cm^-1^ are associated with the vibrations of isopropyl groups. The conversion of NIPAM monomer is also confirmed by the characteristic mode at 1409 cm^-1^, corresponding to the C=C of vinyl groups. After the formation of the crosslinked network, the C=C band disappeared.

**
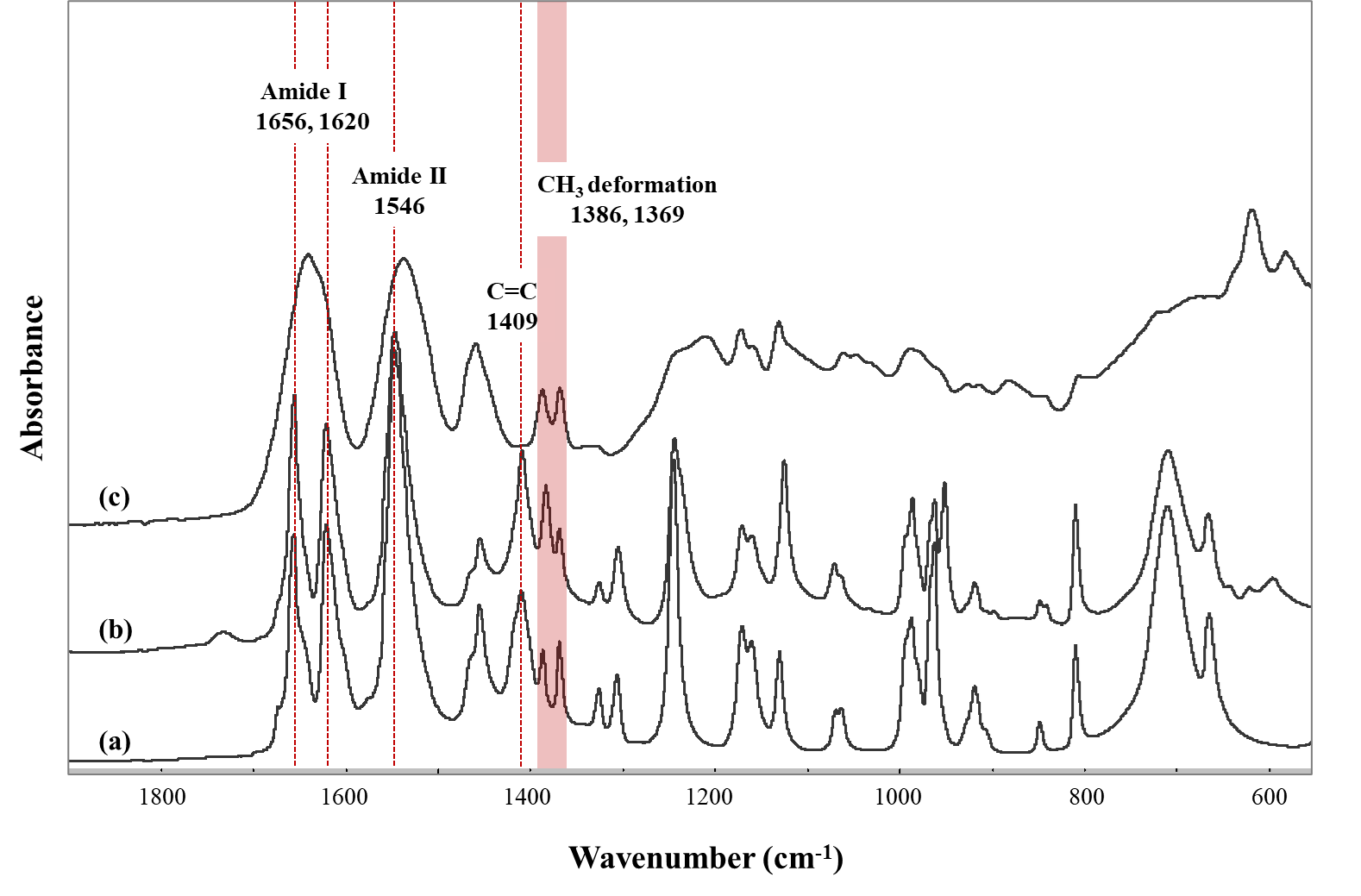
**

**Figure S3** ATR-FTIR spectra of (a) NIPAM monomer, (b) unreacted NIPAM in the supernatant, and (c) polymerized PNIPAM.


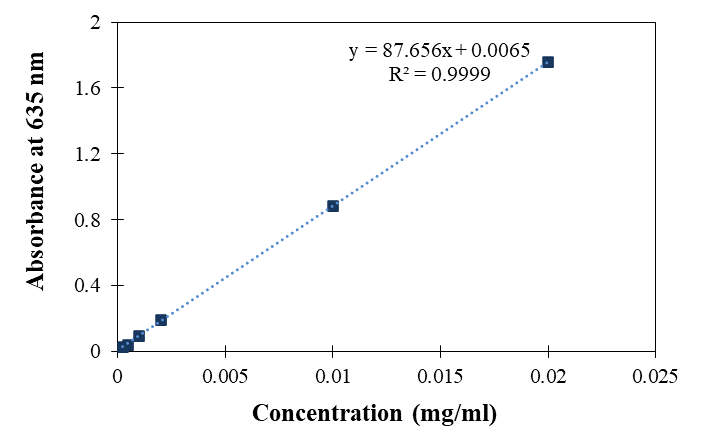


**Figure S4** A calibration curve of Nile Blue A in DI water at pH 7 for *in vitro* release study.


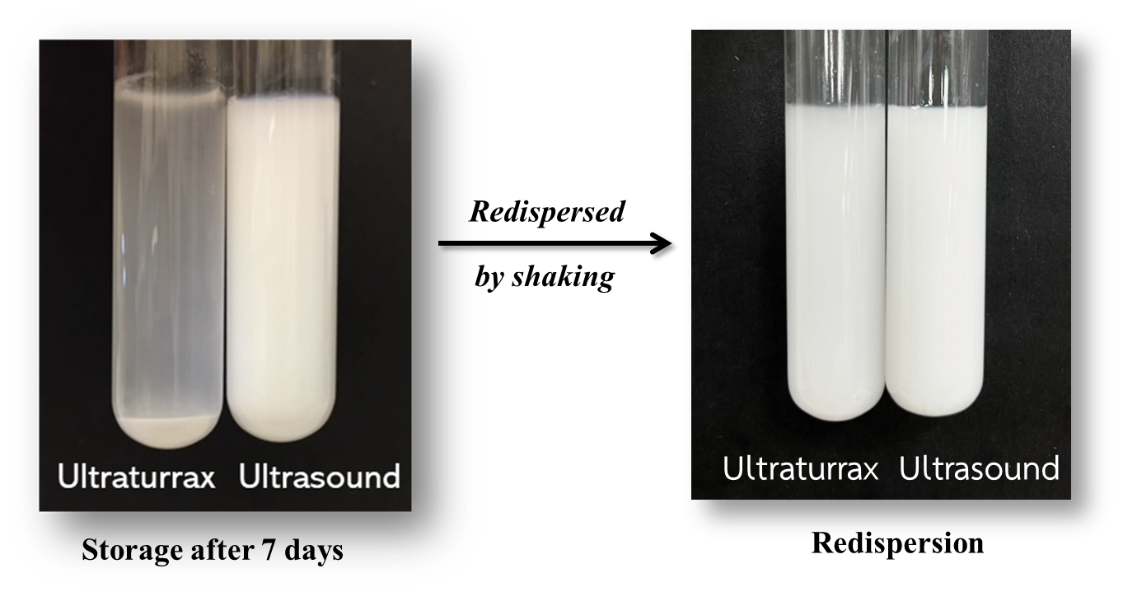


**Figure S5** Physical stability of the nano/microparticles fabricated using UT and US methods in the secondary double emulsion.

**
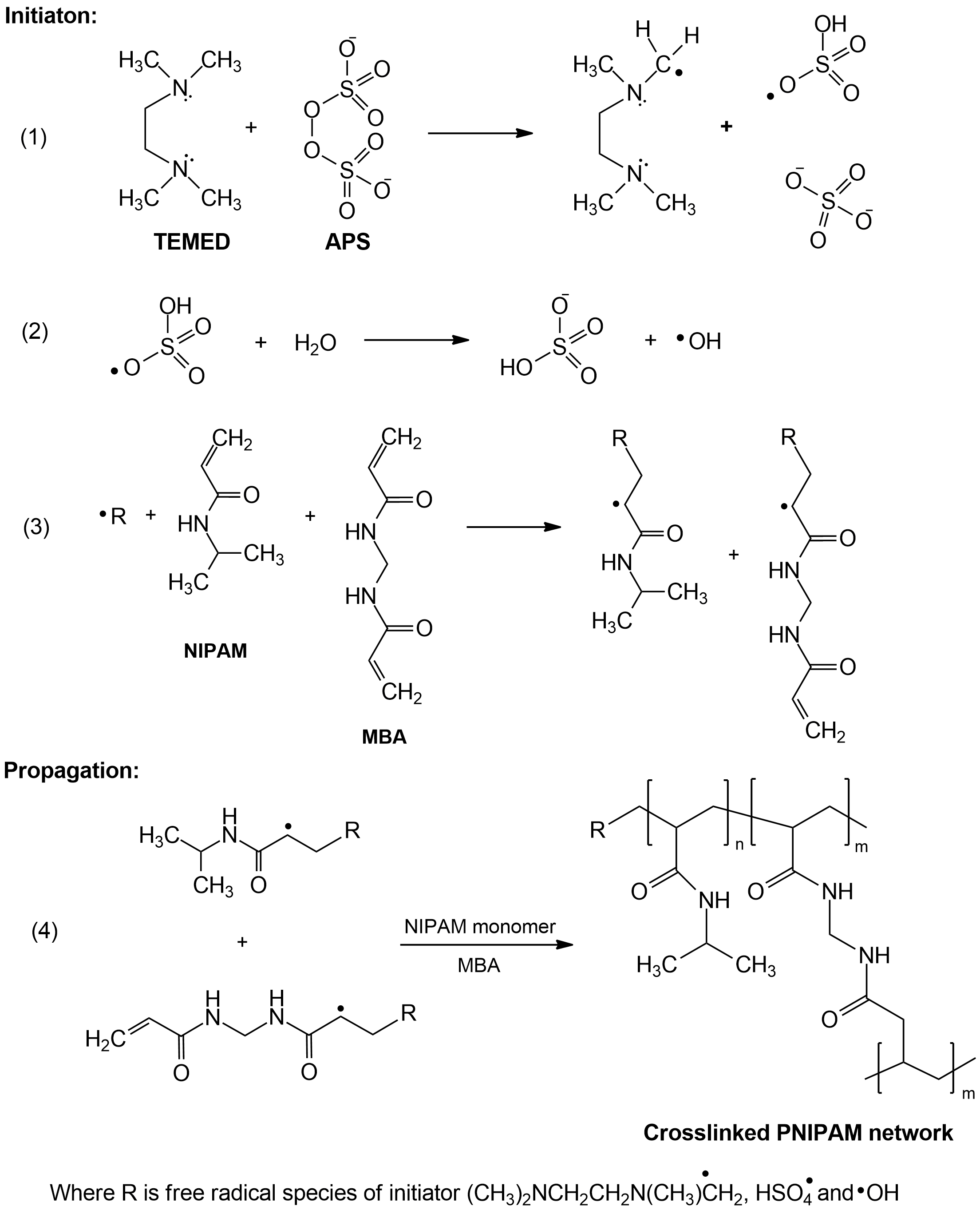
**

**Figure S6** The reaction mechanism of PNIPAM gatekeeper formation by APS/TEMED redox initiated system.


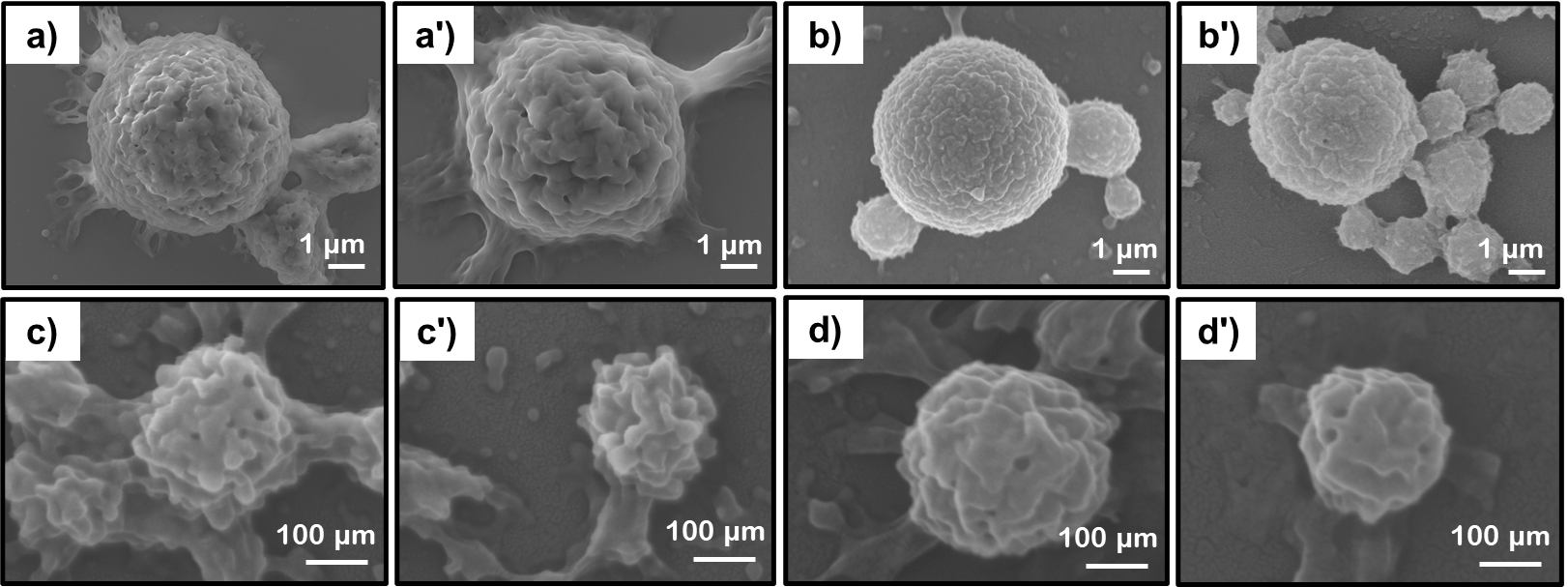


**Figure S7** SEM images of PNIPAM@RS100 particles of (a, a') UT-APS, (b, b') UT-APS/TEMED particles (c, c') US-APS, (d, d') US-APS/TEMED; (a-d) encapsulated NB particles and (a', d') after NB released. The images were taken at 10kX (a-b, a'-b') and 100kX (c-d, c'-d') magnifications.

**Supplementary video:** Time-lapse video illustrating the release behavior of the NB-loaded particles prepared from different initiator systems at 40°C.

The effect of initiator types used in the PNIPAM polymerization on the release characteristic was investigated. In the thermal-initiating system, NB encapsulated in PNIPAM@RS100-APS emulsion is blue due to the acidic environment (pH of 3.4). In contrast, a pink emulsion was observed in the redox-initiating counterpart (PNIPAM@RS100-APS/TEMED) because of the contact between NB and basic TEMED (pH of 8.9). The NB-loaded colloidal particles changed from pink to blue when the temperature was increased to 40°C (above T_VPT_), as the entrapped NB (pink form) was released to a neutral medium (blue form).
